# Diagnostic accuracy of recombinase polymerase amplification-based assays for mosquito-borne virus detection in clinical samples: a systematic review and meta-analysis

**DOI:** 10.3389/fcimb.2026.1911702

**Published:** 2026-09-03

**Authors:** Yonghui Gao, Jihong Chen

**Affiliations:** 1 Department of Healthcare-Associated Infection Control, The People’s Hospital of Baoan Shenzhen, The Second Affiliated Hospital of Shenzhen University, Shenzhen Hospital of Guangdong Provincial People’s Hospital, Hospital Infection Management Quality Control Center of Baoan District, Shenzhen, Guangdong, China; 2 Department of Nephrology, The People’s Hospital of Baoan Shenzhen, The Second Affiliated Hospital of Shenzhen University, Shenzhen Hospital of Guangdong Provincial People’s Hospital, Shenzhen, Guangdong, China

**Keywords:** dengue virus, diagnostic accuracy, meta-analysis, mosquito-borne virus, RPA

## Abstract

**Background:**

Mosquito-borne viral diseases pose an important public health threat due to their frequent outbreaks, similar early clinical manifestations, and potential for rapid transmission. Conventional diagnostic methods, such as virus isolation, serological assays and RT-qPCR, are reliable but may be limited by long turnaround time, cross-reactivity, laboratory equipment requirements and trained operators. Recombinase polymerase amplification (RPA)-based methods have emerged as promising tools for rapid detection of mosquito-borne viruses, but their overall diagnostic accuracy still requires comprehensive evaluation. This meta-analysis aimed to systematically evaluate the diagnostic performance of RPA-based assays for mosquito-borne virus detection.

**Methods:**

A systematic search of PubMed, Embase, Web of Science, Scopus and MEDLINE was conducted using search terms related to mosquito-borne viruses, RPA technology and diagnostic accuracy. Studies reporting sensitivity and specificity or providing sufficient data to construct 2 × 2 contingency tables were included. The methodological quality of included studies was assessed using QUADAS-2. R software was used for statistical analysis. A Bayesian bivariate random-effects model was applied to calculate pooled sensitivity, specificity, positive likelihood ratio (PLR), negative likelihood ratio (NLR) and summary receiver operating characteristic (SROC) curve.

**Results:**

Ten studies involving 13 data sets in total were included in this meta-analysis. The pooled sensitivity and specificity of RPA-based assays were 0.96 (95% CrI: 0.92–0.98) and 0.99 (95% CrI: 0.98–1.00), respectively. The pooled PLR was 146.25 (95% CrI: 35.79–597.57), and the pooled NLR was 0.05 (95% CrI: 0.02–0.10). The area under the SROC curve was 0.987 (95% CrI: 0.972–0.995), suggesting excellent overall diagnostic performance. Subgroup analyses showed that RPA-based assays maintained good diagnostic accuracy in both CRISPR-based and non-CRISPR groups, as well as in DENV and non-DENV subgroups. No significant publication bias was detected by Deeks’ funnel plot asymmetry test.

**Conclusion:**

RPA-based assays showed good diagnostic accuracy for mosquito-borne virus detection, with high pooled sensitivity, specificity and AUC. These findings suggest that RPA-based methods may provide a useful technical choice for rapid case identification and public health response. Nevertheless, the limited number of included studies, methodological limitations and insufficient real-world evidence warrant cautious interpretation. More high-quality prospective studies are still needed to confirm their practical feasibility and application value in clinical and public health settings.

**Systematic review registration:**

https://www.crd.york.ac.uk/prospero/, identifier CRD420261380107.

## Introduction

Mosquito-borne viral diseases, mainly transmitted by *Aedes* and *Culex* mosquitoes, have become an important global public health threat due to their wide geographic distribution, frequent outbreaks, and potential to cause serious clinical and social burden (Pereira et al., 2020; Segura et al., 2021). Dengue virus (DENV), Zika virus (ZIKV), chikungunya virus (CHIKV), West Nile virus (WNV), yellow fever virus, and Japanese encephalitis virus are representative mosquito-borne viruses, which can cause a wide spectrum of diseases ranging from mild febrile illness to severe neurological complications, haemorrhagic manifestations, chronic arthralgia, congenital abnormalities, and even death (Bello et al., 2025; Mbaoma et al., 2025). Currently, the prevention and control of mosquito-borne viral diseases remain challenging due to climate change, urbanization, international travel, population mobility, and the expansion of mosquito habitats, which may further increase the risk of local transmission and cross-regional spread. In recent years, the epidemiological situation has become more complex in China and Southeast Asia. Dengue fever remains the most important vector-borne disease in Southeast Asia and continues to impose a substantial burden on public health systems (Bello et al., 2025). In southern China, dengue outbreaks have repeatedly occurred in areas suitable for *Aedes* mosquitoes, and the suitable habitat of *Aedes albopictus* has shown an expanding trend, suggesting a persistent risk for future dengue transmission (Xie et al., 2025). More importantly, the outbreak of chikungunya fever in Guangdong Province in 2025 highlighted the re-emergence of mosquito-borne viral diseases in southern China and the risk of rapid local transmission in subtropical urban settings (Li et al., 2025; Luo and Sun, 2025; Wang T. et al., 2025). Therefore, the control of mosquito-borne viral diseases depends not only on environmental management and mosquito vector control, but also on early detection of infected cases, timely epidemiological investigation, and appropriate public health interventions. Rapid and accurate diagnosis is essential for medical institutions and public health agencies to identify cases, guide patient management, initiate mosquito-proof isolation or other preventive measures, and reduce the risk of further transmission (Roiz et al., 2018; Salehi et al., 2026).

Conventional laboratory diagnosis of mosquito-borne viral infections mainly includes virus isolation, nucleic acid amplification tests, antigen detection, and serological assays (Johnson et al., 2016; Varghese et al., 2023). Virus isolation can provide direct evidence of viable virus and is useful for virological research, strain characterization, and epidemiological tracing. However, it is usually time-consuming and requires cell culture facilities, appropriate biosafety conditions, and experienced laboratory personnel, which limits its routine application in many clinical and public health institutions (Johnson et al., 2016). Molecular methods, especially RT-PCR and RT-qPCR, are currently widely used for early diagnosis because they can directly detect viral RNA with high sensitivity and specificity during the acute phase of infection (Simo et al., 2023; World Health Organization, 2025). However, these methods still depend on specific instruments, stable laboratory conditions, and professional operation, which may limit their use in primary medical institutions, field investigation, and emergency outbreak response (Varghese et al., 2023; Hauner et al., 2026). Antigen detection, such as NS1 antigen testing for dengue, is relatively rapid and useful during the early stage of infection, but its diagnostic performance may vary with the timing of sample collection, virus serotype, and disease stage (Pillay et al., 2025; World Health Organization, 2025). Serological assays, such as IgM/IgG ELISA, are widely used for later-stage diagnosis and epidemiological investigation, but they are strongly affected by the window period of antibody production and may have cross-reactivity among related arboviruses, especially flaviviruses (Fischer et al., 2021; Chan et al., 2022). In conclusion, although conventional methods are reliable, they cannot always meet the need for rapid and point-of-care testing (POCT). Current laboratory confirmation may result in delayed case identification, epidemiological investigation, isolation of patients or close contacts and mosquito prevention measures, influencing the efficiency of early interruption. Therefore, various rapid diagnostic methods for mosquito-borne viral infections have been continuously developed to improve clinical diagnosis and public health surveillance.

Recent studies have demonstrated the potential of rapid molecular diagnostic methods for the detection of mosquito-borne viruses, especially nucleic acid amplification-based methods, such as loop-mediated isothermal amplification (LAMP), recombinase polymerase amplification (RPA), and CRISPR/Cas-assisted detection systems (Das et al., 2022; Varghese et al., 2023). These methods are usually designed to amplify or recognize specific viral nucleic acid targets within a short time, therefore providing faster and more sensitive evidence of infection than many conventional laboratory methods. Among them, RPA has attracted increasing attention because it can amplify nucleic acids under isothermal conditions at a relatively low temperature, usually around 37–42 °C, by using recombinase, primer, single-stranded DNA binding protein, and strand-displacing polymerase (Daher et al., 2016; Tan et al., 2022). Compared with PCR, RPA does not require repeated thermal cycling, and compared with some other isothermal amplification methods, it has the advantages of rapid reaction, simple operation, low equipment requirement, and better compatibility with portable detection platforms. However, RPA-based assays also have several limitations that should be noted. For example, non-specific amplification may occur when primer or probe design is not optimal. Primer-dependent artifacts and contamination may also lead to false-positive results, especially when amplified products are handled after the reaction (Daher et al., 2016; Tan et al., 2022). Therefore, RPA is often combined with other detection modules, such as reverse transcription, lateral flow strip, fluorescence detection, or CRISPR/Cas systems, to improve its applicability and reduce the influence of some limitations (Zhao et al., 2024; Ji et al., 2025). Among them, the combination of RPA and CRISPR/Cas systems has received special attention. After the reaction of RPA amplification, CRISPR/Cas systems can further recognize specific target sequences through guide RNA and generate detectable signals, which may enhance its analytical specificity and reduce false-positive results caused by non-specific amplification (Ji et al., 2025). Specifically, in this combination, RPA mainly provides products of rapid nucleic acid amplification, while CRISPR/Cas systems provide an additional sequence-specific recognition step after amplification. Only when the amplified product contains the target sequence complementary to the guide RNA can the Cas protein be activated and cleave the reporter probe to generate a detectable signal. Therefore, non-specific amplicons or primer-dependent artifacts are less likely to produce positive signals.

For mosquito-borne viral infections, the application of RPA-based assays may help medical institutions and public health agencies identify infected cases more rapidly, carry out epidemiological investigation earlier, and guide timely mosquito-proof isolation and vector control measures, thereby improving outbreak response and reducing the risk of further transmission. Although many studies have demonstrated the promptness, sensitivity and applicability of RPA-based assays in detecting mosquito-borne viruses, their diagnostic performance may still be affected by assay design, detection platform or validation conditions. Therefore, there remains a notable gap in the comprehensive evaluation of their diagnostic efficiency and accuracy in clinical or public health-related samples (Abd El Wahed et al., 2015; Teoh et al., 2015; Patel et al., 2016; Vasileva Wand et al., 2018). Some studies focused on RT-RPA alone, while others combined RPA with CRISPR/Cas systems or lateral flow detection, which may lead to differences in diagnostic performance across different platforms and target viruses (Yaren et al., 2018; Xi et al., 2019; Athipanyasilp et al., 2026). Therefore, the present study aimed to perform a systematic review and diagnostic meta-analysis to comprehensively evaluate the diagnostic accuracy of RPA-based assays for mosquito-borne viruses. By pooling available diagnostic data, this study further assessed whether RPA-based methods could serve as rapid and reliable tools for case identification, clinical decision-making, and public health surveillance.

## Materials and methods

### Retrieval strategy

This systematic review and diagnostic test accuracy meta-analysis was conducted and reported according to the Preferred Reporting Items for Systematic Reviews and Meta-Analyses of Diagnostic Test Accuracy Studies (PRISMA-DTA) statement. The protocol was registered in PROSPERO (CRD420261380107). Overall, the review was conducted according to the registered protocol, and no changes were made to the main research question, eligibility criteria, index test, reference standard, or primary diagnostic outcomes.

A systematic literature search was performed in PubMed, Embase, Web of Science, Scopus, and MEDLINE to identify eligible studies on RPA-based diagnostic assays for mosquito-borne virus infections. The search terms were constructed using combinations of keywords related to mosquito-borne virus, RPA technology, and diagnostic accuracy, including: (“Dengue” OR “Zika” OR “West Nile” OR “Yellow fever” OR “Japanese encephalitis” OR “Chikungunya” OR “mosquito-borne virus” OR “arbovirus”), (“recombinase polymerase amplification” OR “RPA” OR “RT-RPA” OR “reverse transcription recombinase polymerase” OR “recombinase polymerase”), and (“diagnose*” OR “sensitivity” OR “specificity” OR “clinical” OR “validation” OR “accuracy”). All retrieved records were imported into NoteExpress software for literature management and duplicate removal.

### Inclusion and exclusion criteria

Based on the PICOS framework (Amir-Behghadami and Janati, 2020), studies were eligible for inclusion if they met the following criteria: (1) Population (P): clinical specimens or samples from suspected mosquito-borne virus infections, including dengue virus, Zika virus, West Nile virus, yellow fever virus, Japanese encephalitis virus, CHIKV, or other mosquito-borne viruses; (2) Index test (I): recombinase polymerase amplification-based assays, including RPA, RT-RPA, or related reverse-transcription RPA platforms; (3) Comparator (C): conventional reference or comparator methods, such as RT-qPCR, conventional PCR, virus isolation, serological assays, or other established diagnostic methods; (4) Outcomes (O): studies reporting diagnostic accuracy outcomes, including sensitivity and specificity, or providing sufficient data to construct a 2 × 2 contingency table; and (5) Study design (S): diagnostic accuracy studies or technical validation studies involving clinical samples. Reviews, conference abstracts, case reports, editorials, letters, animal-only studies, and studies without extractable diagnostic accuracy data were excluded.

### Data extraction and quality assessment

The following information was extracted independently by two researchers using a pre-designed Excel spreadsheet: first author, publication year, country, study design, sample type, reference standard, assay type, CRISPR/Cas type if applicable, true positives (TP), false positives (FP), false negatives (FN), and true negatives (TN). For studies reporting separate diagnostic results for different viral serotypes using different primer sets, target sequences and non-overlapping samples, each eligible 2 × 2 table was extracted as an independent data set. Disagreements during data extraction were resolved by discussion. The methodological quality of the included studies was assessed using the Quality Assessment of Diagnostic Accuracy Studies-2 (QUADAS-2) tool. The risk of bias was evaluated in four domains: patient selection, index test, reference standard, and flow and timing. Applicability concerns were assessed in three domains: patient selection, index test, and reference standard. Each domain was judged as “low”, “high”, or “unclear” according to the information reported in the original studies. Review Manager 5.4.0 was used to generate the quality assessment plots.

### Statistical analysis

Statistical analysis was performed using R software (version 4.5.2). A Bayesian bivariate random-effects model implemented in the meta4diag package was used to estimate pooled sensitivity, specificity, positive likelihood ratio (PLR), and negative likelihood ratio (NLR), along with their corresponding 95% credible intervals (CrIs). The summary receiver operating characteristic (SROC) curve and crosshair plot were generated to evaluate the overall diagnostic performance. Threshold effect was assessed by calculating the Spearman correlation between sensitivity and false-positive rate, and by visual inspection of the SROC curve. Heterogeneity was evaluated by the homogeneity test for sensitivity and using Cochran’s Q test and the *I²* statistic based on diagnostic odds ratio (DOR).The variability of individual sensitivity and specificity estimates was also reported. These analyses were performed using the mada package. Sensitivity analysis was conducted using a leave-one-out approach to assess the robustness of the results. In addition, to evaluate the potential influence of multiple data sets from the same study, an additional conservative sensitivity analysis was performed by retaining only one serotype-specific data set from Zhong_2025 at a time and removing the other three. Clinical applicability was evaluated using a Fagan nomogram based on likelihood ratios. Publication bias was assessed using Deeks’ funnel plot asymmetry test implemented via weighted linear regression. All figures were generated using R.

## Results

### Literature screening process

A total of 444 records were identified through systematic database searching, including PubMed (43), Embase (56), Web of Science (100), Scopus (212) and MEDLINE (33). No additional records were identified through other sources. After duplicate removal, 242 records remained for title and abstract screening. Of these, 210 records were excluded because they were irrelevant to the research question, did not involve mosquito-borne virus diagnosis, did not evaluate recombinase polymerase amplification-based assays, or lacked diagnostic accuracy information.

**Figure 1:**
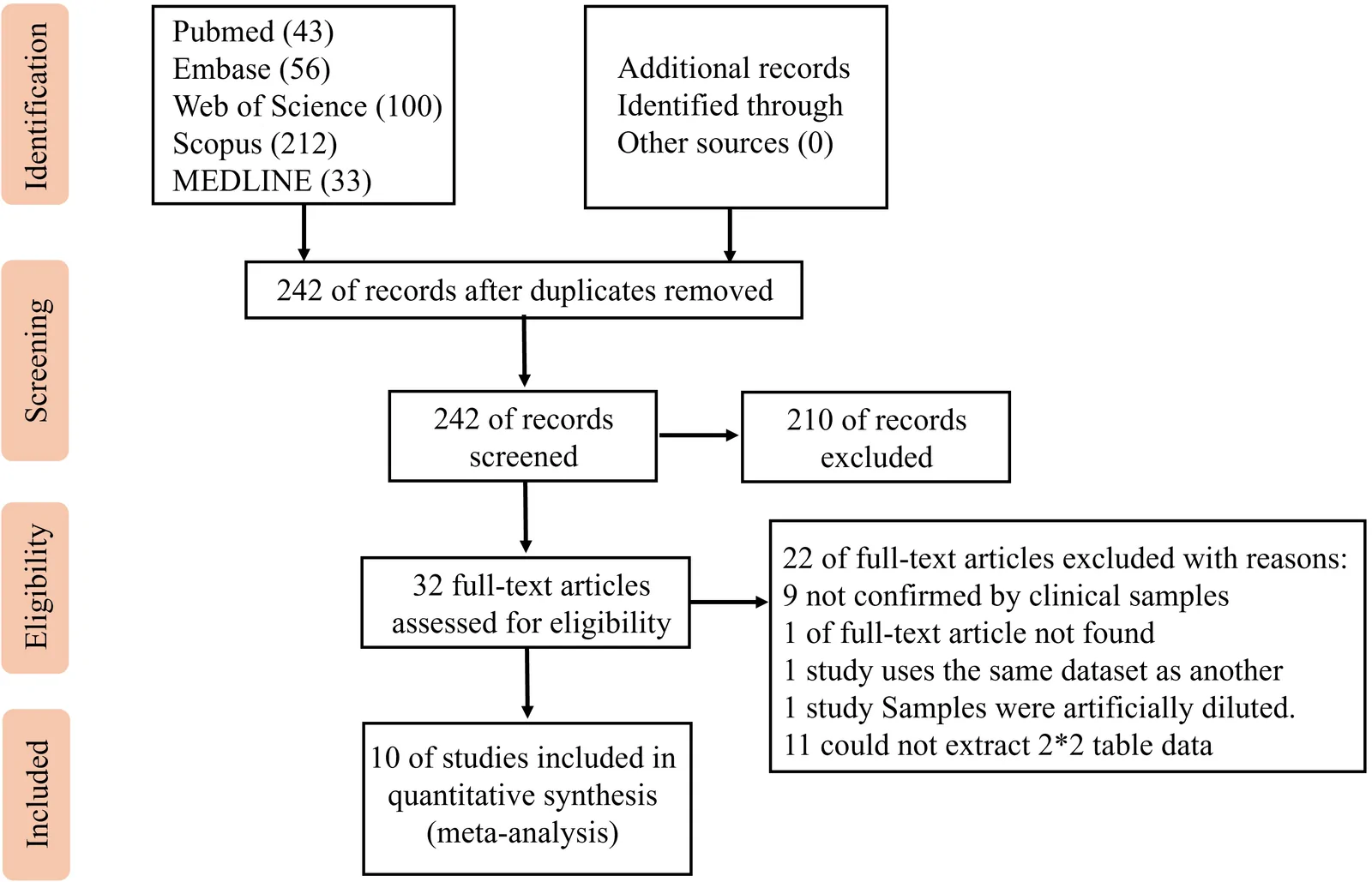
Study flow diagram.

The full texts of 32 potentially eligible articles were then assessed. Twenty-two articles were excluded for the following reasons: nine did not include clinical sample validation, one full-text article could not be retrieved, one study used the same dataset as another eligible study, and eleven studies did not provide sufficient data to construct a 2 × 2 contingency table. Finally, 10 studies were included in the quantitative synthesis and meta-analysis (**Figure 1**) (Teoh et al., 2015; Abd El Wahed et al., 2017; Myhrvold et al., 2018; Vasileva Wand et al., 2018; Xi et al., 2019; Dieng et al., 2020; Tomar et al., 2021; Bhardwaj et al., 2024; Zhong et al., 2025; Athipanyasilp et al., 2026).

### Characteristics of included studies

A total of 13 data sets were extracted from the 10 articles and summarized, as shown in **Table 1**. The included studies were published between 2015 and 2026 and were conducted in different countries or regions, including Malaysia, China, the United Kingdom, Thailand, India, the United States/Brazil/Honduras, Senegal, and Brazil. The target viruses included dengue virus, Zika virus, West Nile virus, and chikungunya virus.

**Table 1:** The detailed information of the included studies.

Author & Year	Country	Study design^a^	Sample type^b^	Reference standard	Type of RPA	TP	FP	FN	TN
Teoh et al. (2015) ^d^	Malaysia	Prospective	Serum	RT-qPCR	RT-RPA	47	3	14	139
Xi et al. (2019) ^d^	China	Prospective	Serum	RT-qPCR	RT-RPA	43	0	0	77
Vasileva Wand et al. (2018) ^d^	United Kingdom	Prospective	Urine, semen, serum, whole blood	RT-qPCR	RT-RPA	25	0	5	25
Athipanyasilp et al. (2026)	Thailand	Diagnostic	Plasma	RT-qPCR	CRISPR-Cas13a-RPA	71	0	2	73
Bhardwaj et al. (2024)	India	Diagnostic	Serum	RT-qPCR	CRISPR-Cas12a-RPA	16	0	0	77
Myhrvold et al. (2018)	USA/Brazil/Honduras	Diagnostic	Serum	RT-qPCR	CRISPR-Cas13a-RPA	10	0	0	6
Zhong et al. (2025)-1 ^c^	China	Diagnostic	Plasma	RT-qPCR	CRISPR-Cas13a-RPA	39	1	1	44
Zhong et al. (2025)-2	China	Diagnostic	Plasma	RT-qPCR	CRISPR-Cas13a-RPA	38	2	2	48
Zhong et al. (2025)-3	China	Diagnostic	Plasma	RT-qPCR	CRISPR-Cas13a-RPA	42	3	3	52
Zhong et al. (2025)-4 ^c^	China	Diagnostic	Plasma	RT-qPCR	CRISPR-Cas13a-RPA	39	1	1	49
Tomar et al. (2021) ^d^	India	Prospective	Serum and plasma	RT-qPCR	RT-RPA	24	0	1	85
Dieng et al. (2020) ^d^	Senegal	Prospective	Serum	RT-qPCR	RT-RPA	3	0	0	101
Abd El Wahed et al. (2017) ^d^	Brazil	Diagnostic	Urine	RT-qPCR	RT-RPA	23	0	2	9

a. “Prospective” indicates prospective sample collection, while “Diagnostic” refers to diagnostic accuracy or clinical validation studies.

b. Some studies included multiple specimen types.

c. Zhong_2025–1 to Zhong_2025–4 represent different subgroups extracted from the same study. These four data sets corresponded to four DENV serotype-specific assays. They used different primer sets and target sequences, and were reported as separate 2 × 2 contingency tables in the original article.

d. RT-RPA indicates assays without CRISPR/Cas components.

The sample types mainly included serum, plasma, urine, whole blood, semen, and mixed clinical specimens. qPCR or RT-qPCR was used as the reference standard in all included studies. Among the 13 extracted data sets, 6 evaluated RT-RPA-based assays without CRISPR/Cas components, while 7 evaluated RPA combined with CRISPR/Cas systems. The extracted diagnostic data included TP, FP, FN, and TN, which were used to construct 2 × 2 contingency tables for quantitative analysis.

### Quality evaluation

**Figure 2:**
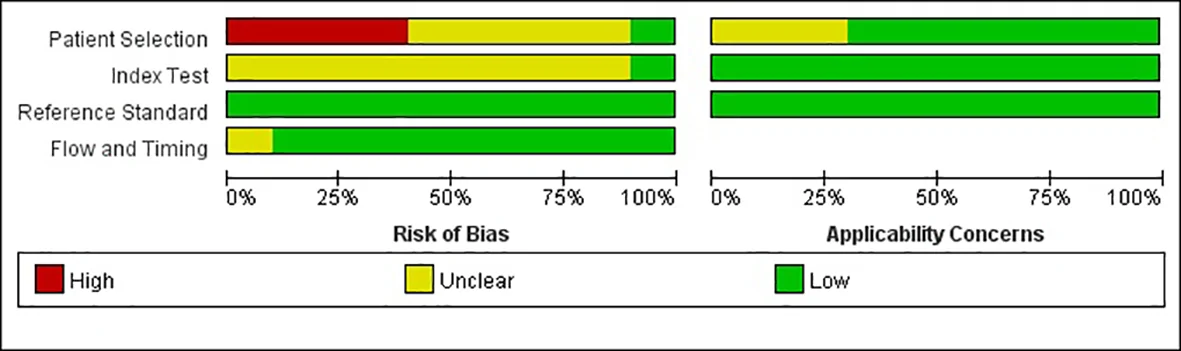
The summary of the risk of bias and applicability concerns of the included studies.

**Figure 3:**
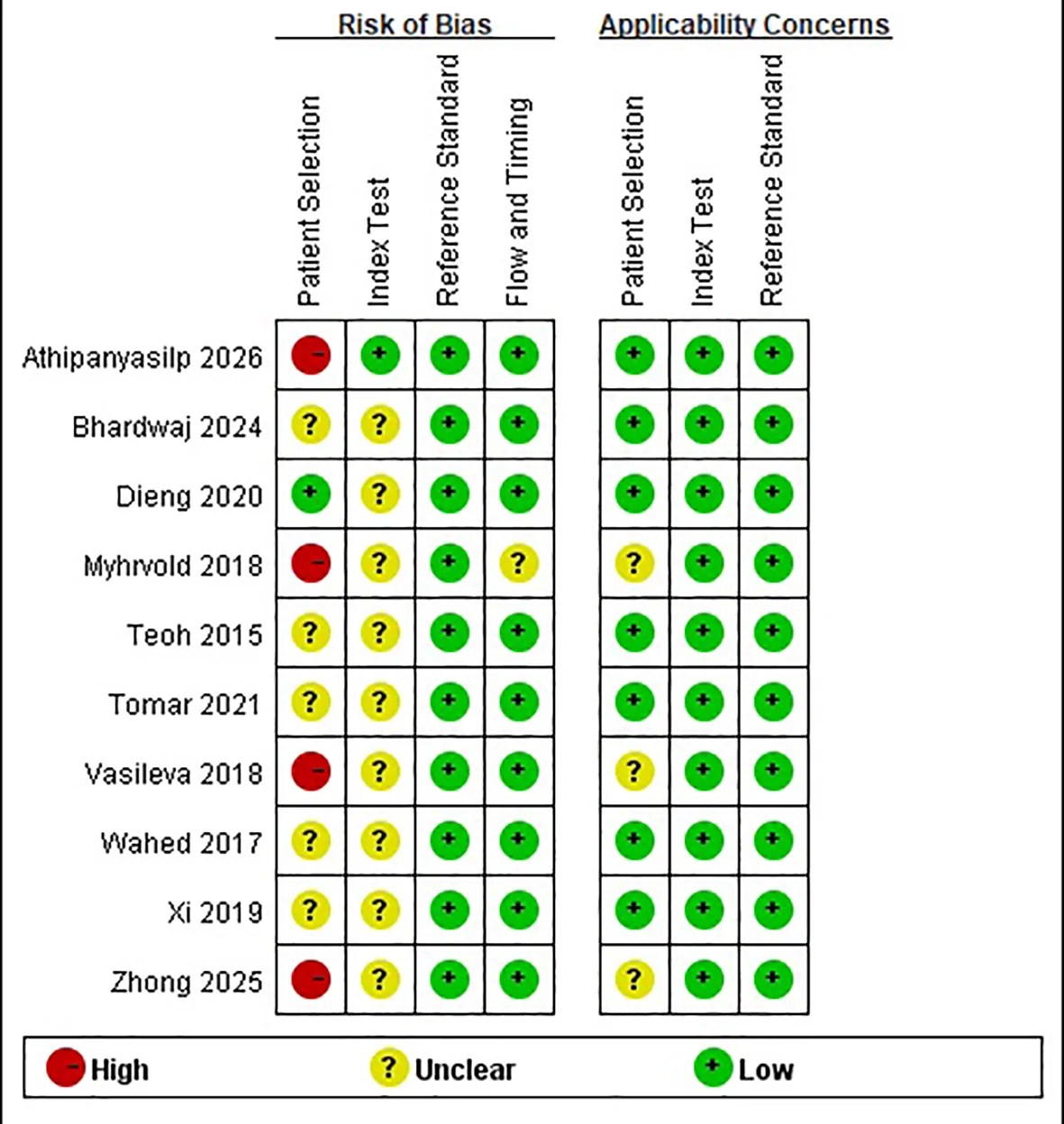
Quality evaluation of the individual studies.

Review Manager 5.4.0 was used to produce the quality assessment plots (**Figures 2**, **3**). In the domain of patient selection, 4 studies were considered to have a high risk of bias because they did not avoid a case–control design or used selected validation samples, and 5 studies had an unclear risk because they did not clearly report whether the samples were enrolled consecutively or randomly. For the index test, 9 studies were judged as having an unclear risk of bias, mainly because they did not clearly state whether the index test results were interpreted without knowledge of the reference standard results. In the domain of reference standard, all included studies were considered to have a low risk of bias because RT-qPCR or real-time RT-PCR was used as the reference method. Regarding flow and timing, 1 study had an unclear risk of bias due to insufficient information on the testing flow, while the remaining studies were judged as low risk. For applicability concerns, most studies showed low concern in the domains of index test and reference standard, while 3 studies had unclear concern in patient selection because of the use of selected sample panels or mixed validation samples.

### Publication bias

**Figure 4:**
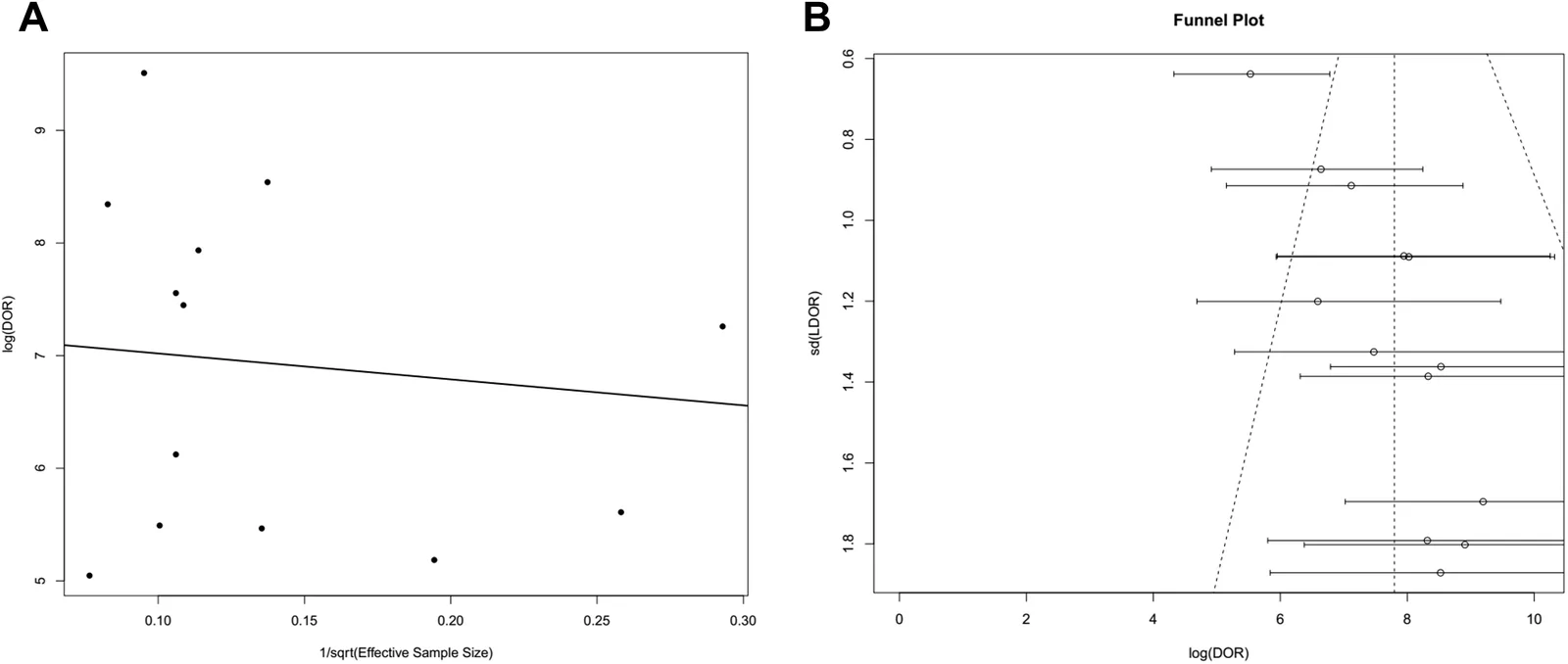
Publication bias of the included data sets. **(A)** Deeks’ funnel plot for assessment of publication bias. The Deeks’ funnel plot displays the relationship between the log diagnostic odds ratio [log(DOR)] and the inverse square root of the effective sample size (1/√ESS). Each point represents an individual data set. The regression line was used to evaluate funnel plot asymmetry (slope = −2.303, p = 0.861). **(B)** Conventional funnel plot for assessment of publication bias. The funnel plot shows the relationship between the log diagnostic odds ratio [log(DOR)] and its standard deviation [sd(LDOR)] across the included studies. Each point represents an individual data set.

Publication bias and small-study effects were primarily assessed using Deeks’ funnel plot asymmetry test. No significant small-study effect was detected (slope = −2.303, p = 0.861) (**Figure 4A**). Visual inspection of the Deeks’ funnel plot also showed no obvious asymmetry. In addition, a conventional funnel plot based on log(DOR) against sd(LDOR) was provided for visual reference and showed a generally symmetrical distribution of the included studies (**Figure 4B**).

### Overall diagnostic accuracy

**Figure 5:**
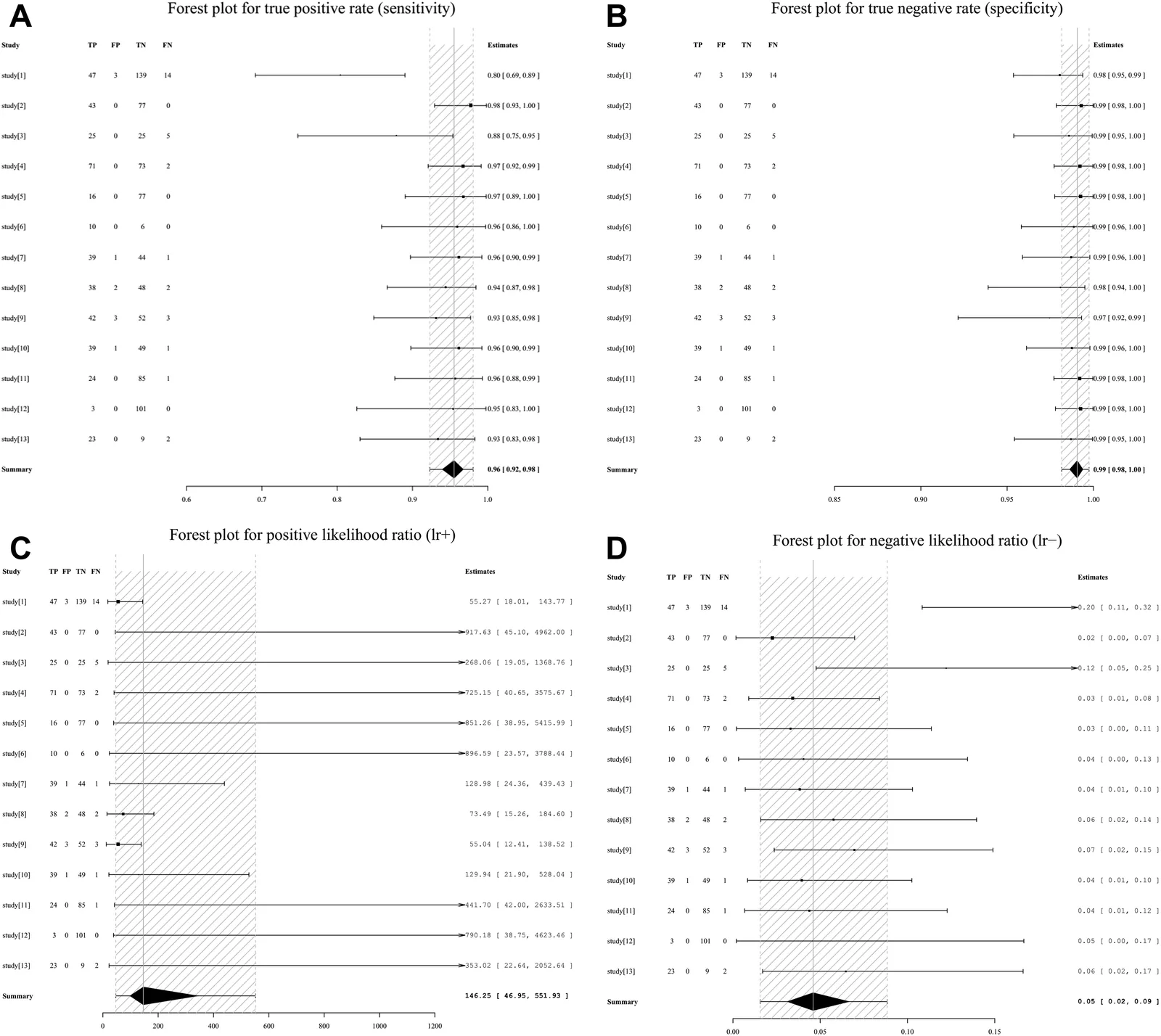
Forest plots for the pooled sensitivity **(A)**, specificity **(B)**, PLR **(C)** and NLR **(D)** of the included studies.

The pooled sensitivity across the included data sets was 0.96 (95% CrI: 0.92–0.98), while the pooled specificity was 0.99 (95% CrI: 0.98–1.00). The pooled positive likelihood ratio (LR+) was 146.25 (95% CrI: 35.79–597.57), and the pooled negative likelihood ratio (LR−) was 0.05 (95% CrI: 0.02–0.10). Across individual data sets, sensitivity estimates ranged from 0.77 to 1.00, while specificity estimates ranged from 0.94 to 1.00. The LR+ values varied substantially among studies, whereas LR− values remained low across most included studies (**Figure 5**).

### SROC curve and crosshair plot

**Figure 6:**
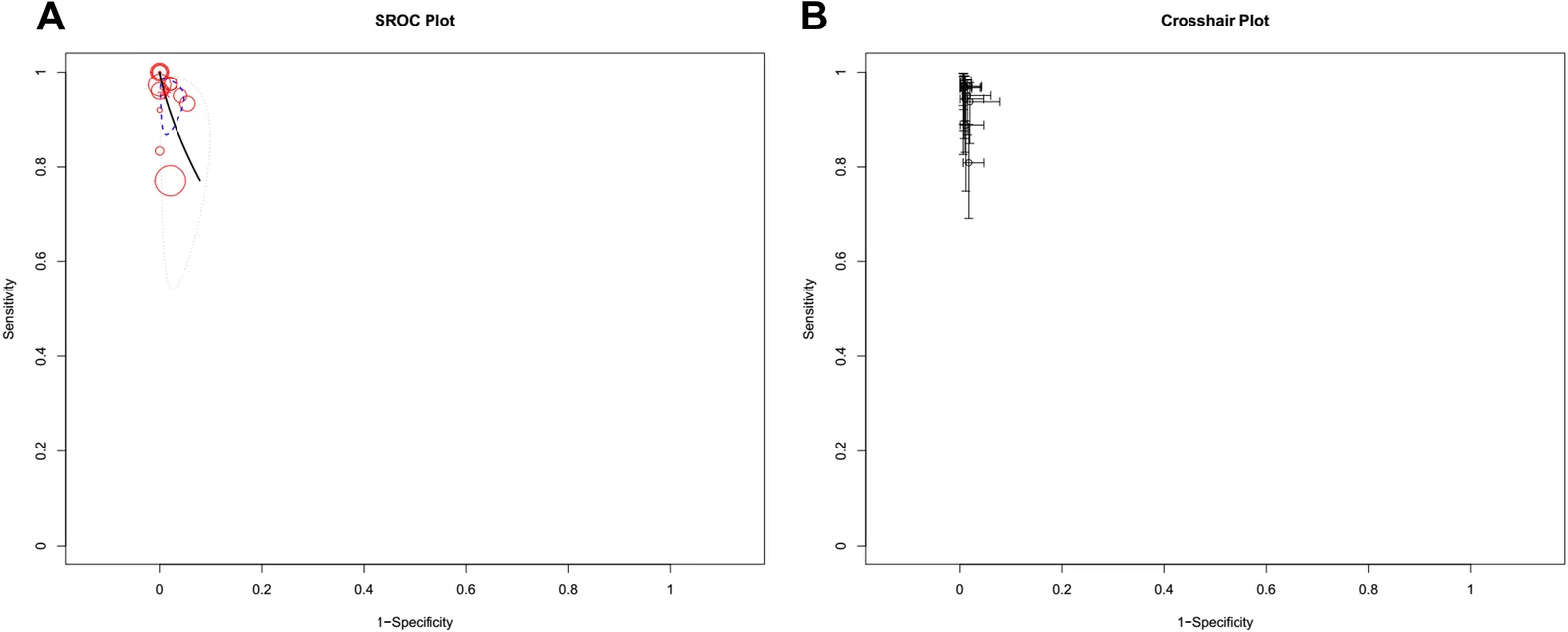
SROC curve and crosshair plot of the included studies. **(A)** The SROC curve summarizes the overall diagnostic performance across studies, with sensitivity plotted against 1−specificity. Each circle represents an individual data set, with the size proportional to the study weight. The solid curve indicates the summary ROC curve derived from the bivariate model. The central point represents the pooled estimate, and the surrounding region indicates the confidence or prediction region. **(B)** Each point in the crosshair plot represents an individual data set, with horizontal and vertical lines indicating the corresponding 95% confidence intervals for sensitivity and specificity, respectively. The distribution of studies in the receiver operating characteristic space is shown, with sensitivity plotted against 1−specificity.

The SROC curve summarizes the trade-off between sensitivity and specificity across the included studies, with an area under the curve (AUC) of 0.987 (95% CrI: 0.972–0.995) (**Figure 6A**). Meanwhile, in the crosshair plot, each study is represented by a point with horizontal and vertical lines indicating the corresponding 95% confidence intervals for specificity and sensitivity, respectively. Most studies are distributed in the upper-left region of the ROC space, corresponding to high sensitivity and low 1−specificity. The spread of confidence intervals varies across studies, with some studies showing wider intervals in either sensitivity or specificity (**Figure 6B**).

### Sensitivity analysis

To further test the robustness of the findings in this study, a sensitivity analysis was performed using the leave-one-out method. The results showed that after excluding each individual data set one at a time, there were no obvious fluctuations in the pooled sensitivity, pooled specificity, positive likelihood ratio (PLR), negative likelihood ratio (NLR), and diagnostic odds ratio (DOR). The pooled sensitivity ranged from 0.946 to 0.962, the pooled specificity ranged from 0.989 to 0.993, the PLR ranged from 86.000 to 137.286, the NLR ranged from 0.038 to 0.055, and the DOR ranged from 1575.074 to 3495.505 (**Table 2**). These results suggested that the overall results of this study were not excessively influenced by any single data set, and the conclusions are relatively robust.

**Table 2:** Leave-one-out sensitivity analysis by omitting one data set at a time.

Data set omitted	Sensitivity	Specificity	PLR	NLR	DOR
Teoh-2015	0.961	0.993	137.286	0.039	3495.505
Xi-2019	0.946	0.989	86.000	0.055	1575.074
Vasileva-2018	0.962	0.991	106.889	0.038	2787.550
Athipanyasilp-2026	0.953	0.989	86.636	0.048	1823.050
Bhardwaj-2024	0.951	0.989	86.455	0.050	1744.970
Myhrvold-2018	0.952	0.990	95.200	0.048	1963.500
Zhong-2025-1	0.953	0.992	119.125	0.047	2514.298
Zhong-2025-2	0.957	0.993	136.714	0.043	3157.146
Zhong-2025-3	0.958	0.993	136.857	0.042	3235.694
Zhong-2025-4	0.953	0.992	119.125	0.047	2514.298
Tomar-2021	0.955	0.989	86.818	0.046	1908.071
Dieng-2020	0.953	0.989	86.636	0.048	1823.050
Wahed-2017	0.959	0.991	106.556	0.041	2575.526

What’s more, considering that four serotype-specific data sets were extracted from Zhong_2025, we further performed an additional conservative sensitivity analysis by retaining only one of these four data sets at a time and removing the other three. In this analysis, 10 data sets were included each time. The pooled sensitivity ranged from 0.948 to 0.957, and the pooled specificity ranged from 0.996 to 0.997, which were close to the main analysis (**Supplementary Table 1**). These results suggested that the overall conclusion was not mainly driven by Zhong_2025.

### Threshold effect and heterogeneity

**Figure 7:**
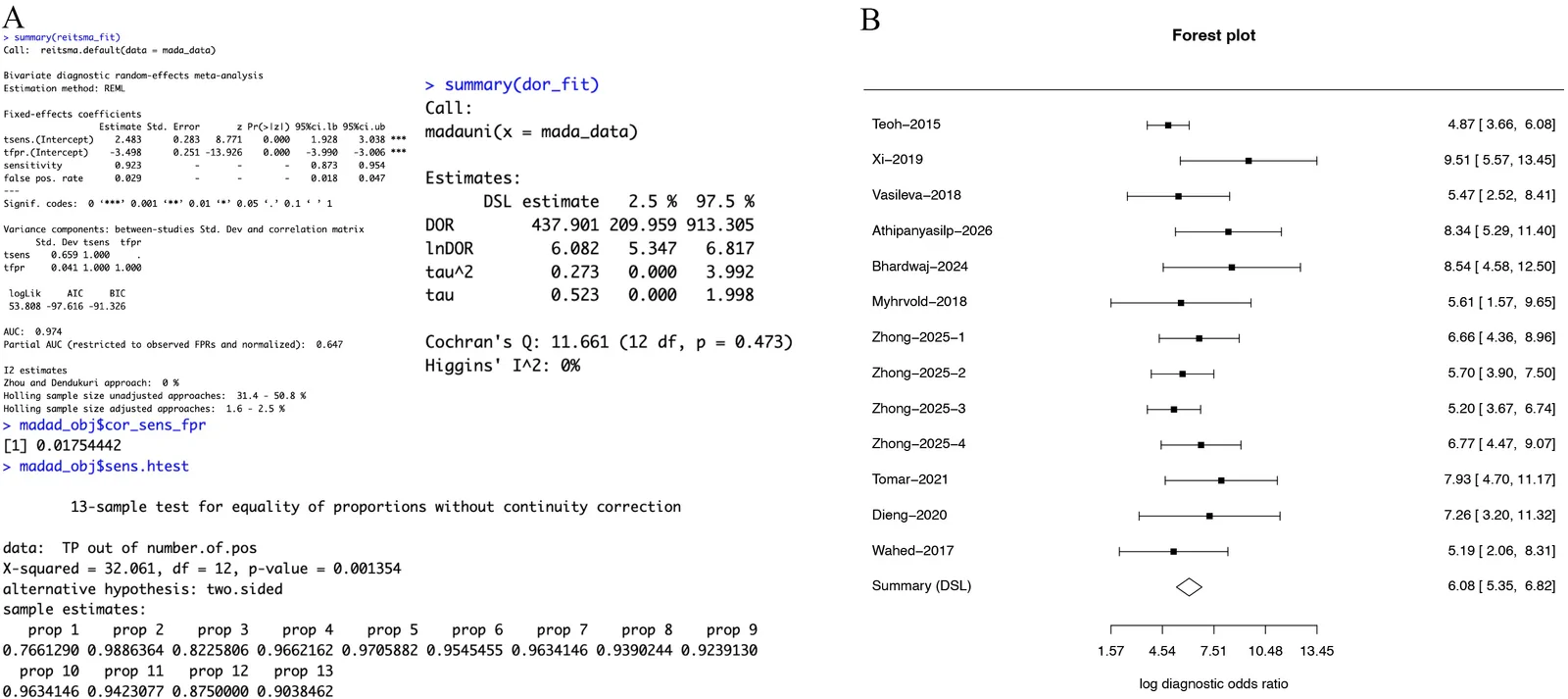
Threshold effect and heterogeneity analysis. **(A)** Results of threshold effect and heterogeneity analysis using the mada package. The correlation coefficient between sensitivity and false-positive rate was used to evaluate the threshold effect. The homogeneity test for sensitivity and the DOR-based heterogeneity analysis was also shown. **(B)** Forest plot of log diagnostic odds ratio. Each square represents the log DOR of an individual data set, and the horizontal line represents its 95% confidence interval. The diamond represents the pooled estimate. DOR, diagnostic odds ratio.

The threshold effect was evaluated using the mada package. The correlation coefficient between sensitivity and false-positive rate was 0.018, suggesting no obvious threshold effect (**Figure 7A**). In addition, the SROC curve showed no typical “shoulder-arm” pattern, which further supported the absence of an obvious threshold effect (**Figure 6A**). Then, heterogeneity was further evaluated among the included data sets. The homogeneity test for sensitivity showed significant heterogeneity across data sets (χ² = 32.061, df = 12, *p* = 0.001) (**Figure 7A**). Indeed, the individual sensitivity estimates ranged from 0.77 to 1.00, while the individual specificity estimates ranged from 0.94 to 1.00, indicating that variability existed across the included data sets. In the DOR-based heterogeneity analysis, no significant heterogeneity was detected, with Cochran’s Q = 11.661, df = 12, *p* = 0.473, and Higgins’ *I²* = 0% (**Figure 7A**). The forest plot of log diagnostic odds ratio also visually supported this result, showing a relatively stable pooled estimate across data sets (**Figure 7B**). However, because DOR combines sensitivity and specificity into one single indicator, this result was considered supplementary and could not exclude the variability observed in sensitivity. Therefore, though there was no obvious heterogeneity observed based on DOR, the pooled diagnostic estimates should be interpreted with caution.

### Clinical applicability

**Figure 8:**
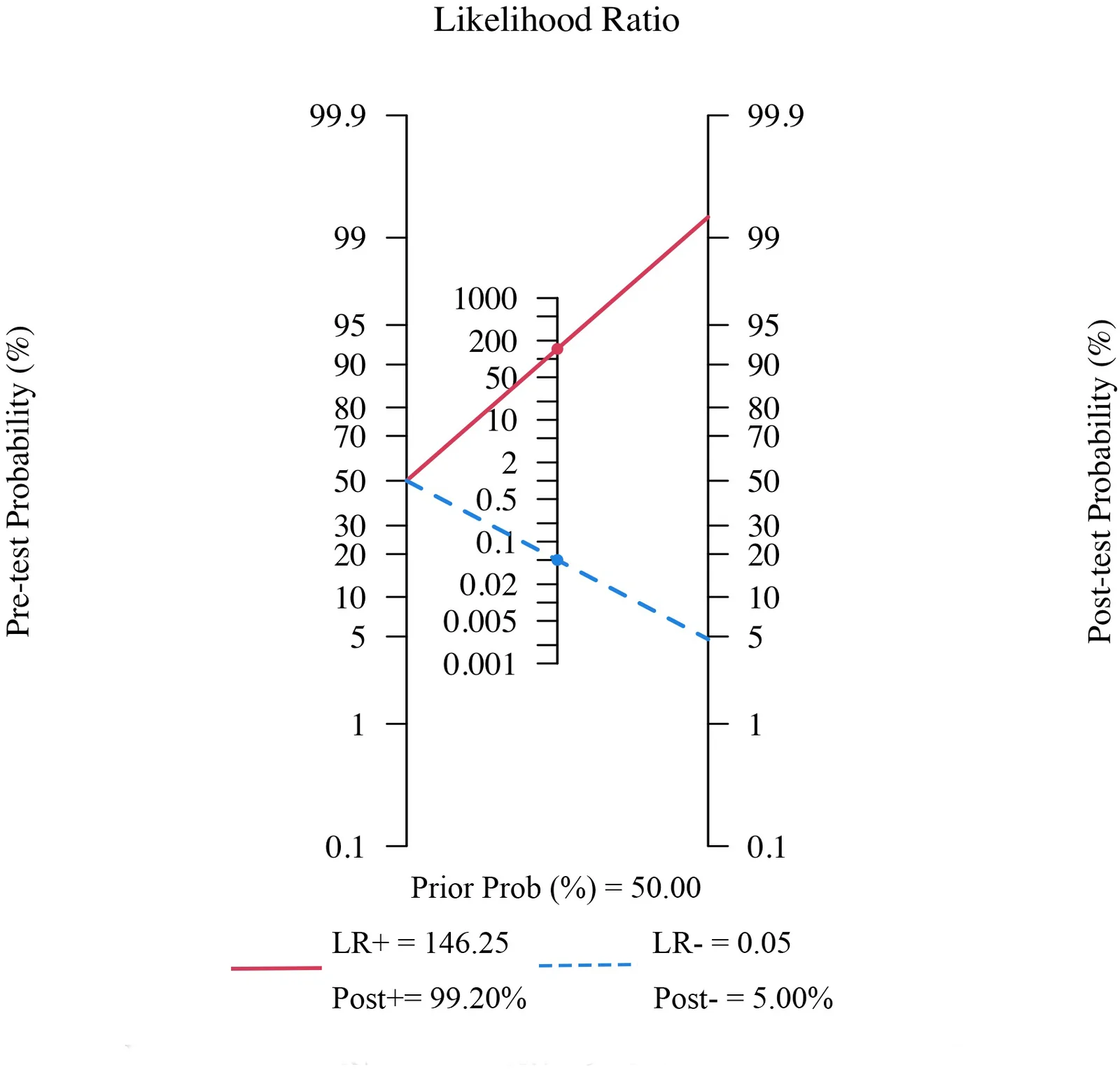
Fagan’s probability plot.

The Fagan nomogram showed that at a pretest probability of 50%, a positive test result increased the probability of disease to approximately 99.20% when the pooled positive likelihood ratio (LR+) was 146.25, whereas a negative test result decreased the probability of disease to approximately 5% when the pooled negative likelihood ratio (LR−) was 0.05 (**Figure 8**).

### Subgroup analysis

**Figure 9:**
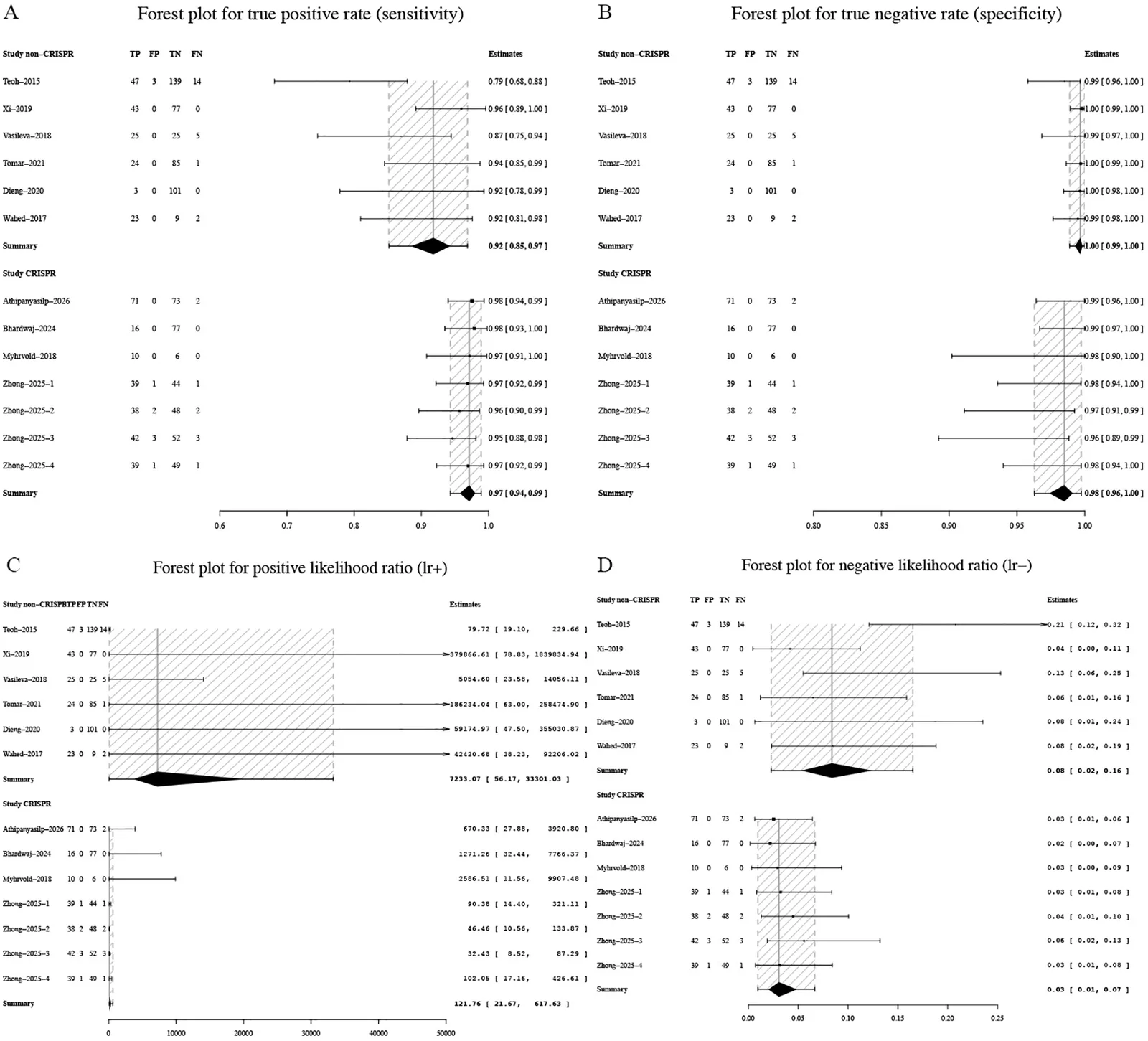
Forest plots for the pooled sensitivity **(A)**, specificity **(B)**, PLR **(C)** and NLR **(D)** of the included studies stratified by assay platform (CRISPR-based vs non-CRISPR-based).

In the subgroup analysis stratified by assay platform, 7 data sets were included in the CRISPR-based group and 6 data sets were included in the non-CRISPR group. The pooled sensitivity of the CRISPR group was 0.971 (95% CrI: 0.943–0.989), which was higher than that of the non-CRISPR group (0.918, 95% CrI: 0.852–0.969). In contrast, the pooled specificity of the non-CRISPR group was 0.997 (95% CrI: 0.989–1.000), slightly higher than that of the CRISPR group (0.985, 95% CrI: 0.963–0.997) (**Table 3**). These results showed numerical differences between the CRISPR-based and non-CRISPR groups in the included data sets. However, this subgroup analysis was exploratory and should not be interpreted as direct comparative evidence, because the number of data sets was limited and potential confounding factors, such as virus type, sample type and study design, could not be fully controlled. (**Figure 9**).

**Table 3:** Subgroup results of the diagnostic meta-analysis by assay platform and virus type.

Subgroup variable	Subgroup	No. of data sets	Pooled sensitivity	95% CrI (Sensitivity)	Pooled specificity	95% CrI (Specificity)
Assay platform	CRISPR	7	0.971	0.943-0.989	0.985	0.963-0.997
Assay platform	Non- CRISPR	6	0.918	0.852-0.969	0.997	0.989-1.000
Virus type	DENV	7	0.954	0.904-0.986	0.982	0.969-0.992
Virus type	Non-DENV	6	0.955	0.899-0.987	1.000	1.000-1.000

**Figure 10:**
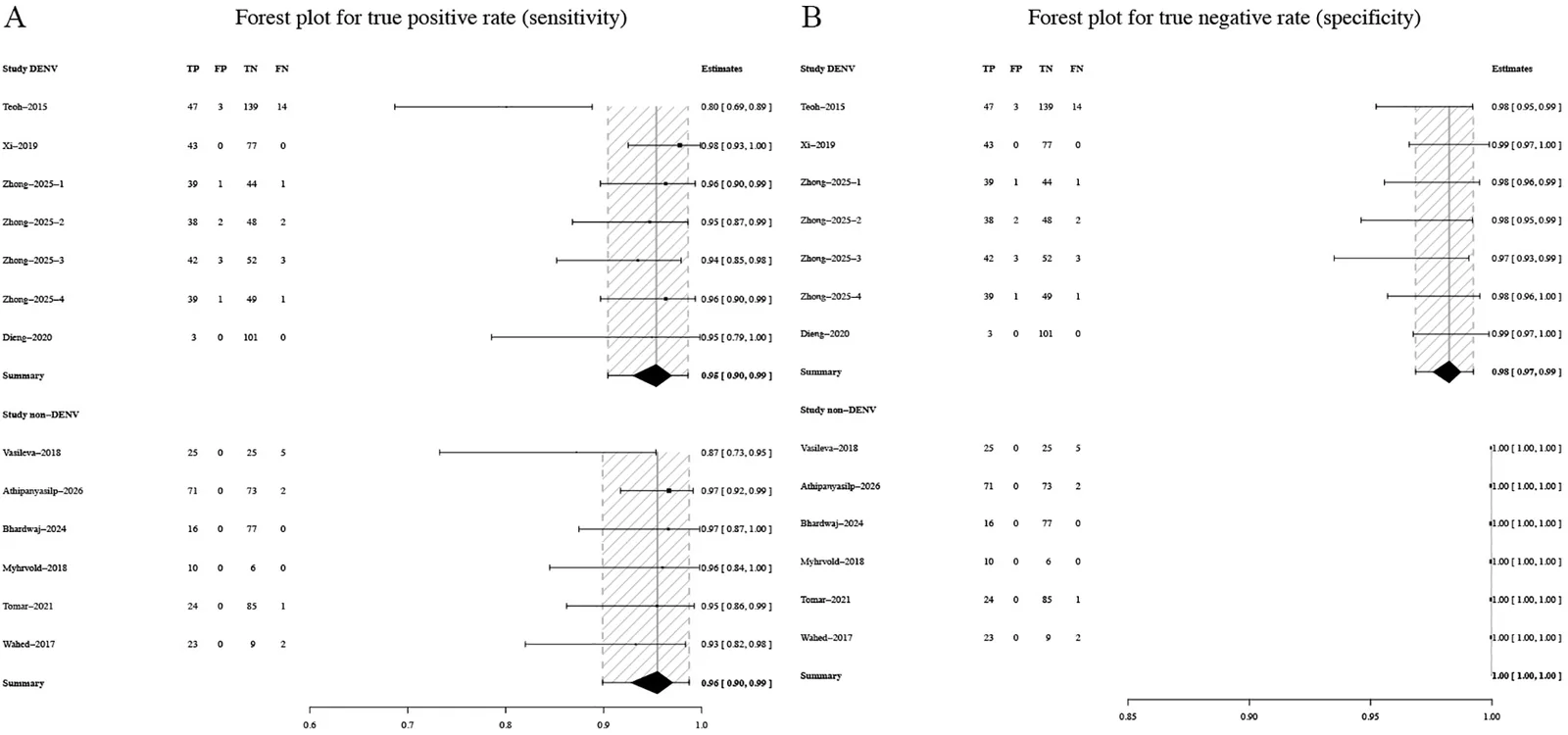
Forest plots for the pooled sensitivity **(A)**, specificity **(B)** of the included studies stratified by virus type (DENV vs non-DENV).

We further conducted subgroup analysis based on virus type (DENV and non-DENV subgroups), both subgroups demonstrated high overall diagnostic performance. The DENV subgroup had a pooled sensitivity of 0.954 (95% CrI: 0.904–0.986) and a pooled specificity of 0.982 (95% CrI: 0.969–0.992). The non-DENV subgroup had a pooled sensitivity of 0.955 (95% CrI: 0.899–0.987) and a pooled specificity of 1.000 (**Table 3**). Overall, the DENV and non-DENV subgroups showed similar pooled sensitivity, while the non-DENV subgroup had higher pooled specificity. However, because several data sets in the non-DENV subgroup reported zero false positives, the specificity estimate in this subgroup showed boundary clustering and should be interpreted cautiously. (**Figure 10**).

## Discussion

In the present study, we performed a diagnostic meta-analysis to figure out the diagnostic accuracy of RPA-based assays in detecting mosquito-borne viruses in clinical or public health-related samples. Our results showed that the pooled sensitivity and specificity of the included data sets were 0.96 and 0.99, respectively. Meanwhile, the area under the SROC curve (AUC) was 0.987, suggesting that RPA-based molecular methods achieved excellent diagnostic performance for mosquito-borne virus detection. In addition, the pooled LR+ was 146.25, while the pooled LR− was 0.05, indicating that a positive RPA-based result could strongly support the diagnosis of mosquito-borne viral infection, and a negative result could substantially reduce the probability of infection. Actually, similar results have also been observed in studies evaluating other isothermal amplification methods for mosquito-borne viruses. For example, an RT-LAMP assay for chikungunya virus detection showed 100% clinical sensitivity and 96.72% clinical specificity in patient samples, and a LAMP-based point-of-care system for dengue, chikungunya and Zika virus infections also showed favorable diagnostic performance in the early stage of illness (Silva SJRD. et al., 2019; Kutsuna et al., 2020; Silva et al., 2024). Despite different amplification principles, these rapid molecular methods showed consistently high diagnostic accuracy, indicating that isothermal amplification-based assays may maintain good performance in the detection of mosquito-borne viruses. In this case, RPA-based assays may provide useful diagnostic evidence for medical institutions and public health agencies, especially in early case identification, epidemiological investigation, mosquito-proof isolation and timely vector control, therefore helping to improve outbreak response and reduce the risk of further transmission.

In actual clinical and public health practice, rapid and accurate detection of mosquito-borne viral infections is essential not only for patient management, but also for case reporting, epidemiological investigation and vector control. In our study, the pooled PLR of 146.25 and the NLR of 0.05 indicated strong diagnostic power. Furthermore, the Fagan nomogram indicated that when the pre-test probability was 50%, the post-test probability increased to approximately 99.20% with a positive result, and decreased to approximately 5% with a negative result. These results together indicated that positive results can strongly increase the probability of mosquito-borne viral infection, while negative results can greatly reduce the possibility of infection. Actually, likelihood ratios above 10 and below 0.1 are generally considered to provide strong evidence for ruling in or ruling out a diagnosis, respectively (Deeks and Altman, 2004; Akobeng, 2007). Therefore, these findings allow staff in clinical and public health institutions to directly see the diagnostic value of RPA-based detection methods, enhancing their confidence in early test results, and make timely actions based on the results.

In summary, these findings have several important clinical and public health implications:

1. Earlier case identification and patient management: rapid and accurate detection allows medical institutions to identify suspected mosquito-borne viral infections earlier, guide clinical monitoring and supportive treatment, and reduce diagnostic uncertainty during the acute febrile stage (Harrington et al., 2013; Van Wyk et al., 2025).
2. Timely epidemiological investigation and reporting: early laboratory evidence can help public health agencies initiate case reporting, travel or exposure history investigation, cluster investigation when necessary, and risk assessment more rapidly. Early detection and surveillance have been considered important components in dengue outbreak prediction, detection and response (Harrington et al., 2013; Van Wyk et al., 2025).
3. Improved transmission control: rapid identification of infected cases enables timely mosquito-proof isolation, personal mosquito-bite prevention and targeted vector control, thereby reducing the chance that mosquitoes bite viremic patients and further transmit the virus (Shang et al., 2010; Xu et al., 2026).
4. Public health and economic benefits: earlier diagnosis and more targeted response may reduce unnecessary repeated testing, avoid delayed outbreak recognition, improve the allocation of surveillance and vector control resources, and decrease the social and healthcare burden caused by mosquito-borne viral outbreaks. Integrated strategies including surveillance, case detection, vector control and community-level interventions have been emphasized for dengue, Zika and chikungunya virus infections (Côrtes et al., 2023; Xu et al., 2026).

Meanwhile, compared with conventional PCR-based molecular methods, RPA-based assays have several practical advantages in the rapid detection of pathogens. Although RT-qPCR is still regarded as an important and reliable molecular method, it usually depends on thermal cycling instruments, stable laboratory conditions and trained operators. On the contrary, RPA can amplify nucleic acids under a relatively low temperature without using complex equipment, usually around 37–42 °C, through the cooperation of recombinase, primers, single-stranded DNA binding protein and strand-displacing polymerase (Daher et al., 2016; Tan et al., 2022). These features empower RPA potential for rapid molecular diagnosis in settings where conventional laboratory resources are limited. In the present study, we further divided the included data sets into CRISPR-based and non-CRISPR groups for sub-group analysis. The CRISPR-based group showed a pooled sensitivity of 0.971 and specificity of 0.985, while the non-CRISPR group showed a pooled sensitivity of 0.918 and specificity of 0.997. This result suggested that both types of RPA-based assays achieved good diagnostic performance for mosquito-borne virus detection. The numerically higher pooled sensitivity in the CRISPR-based group may be related to the additional nucleic acid recognition and signal amplification effect of CRISPR/Cas systems, which has also been reported in other CRISPR-based viral diagnostic studies (Myhrvold et al., 2018; Wang Z. et al., 2025). However, this finding should be considered exploratory and interpreted carefully, because the two subgroups were not compared in the same study population, and differences in target viruses, sample types and study designs may also influence the pooled results. Therefore, the result of subgroup analysis should not be simply explained as direct evidence that CRISPR-based assays are superior to non-CRISPR assays. More importantly, both subgroups maintained high sensitivity and specificity, indicating that RPA-based molecular methods may have stable diagnostic potential and could provide a useful technical choice for rapid case identification and public health response in mosquito-borne viral diseases.

Another important finding of this study was that RPA-based assays showed good diagnostic performance in both DENV and non-DENV subgroups. In the present study, the DENV subgroup had a pooled sensitivity of 0.954 and specificity of 0.982, while the non-DENV subgroup had a pooled sensitivity of 0.955 and specificity of 1.000. This result indicated that RPA-based methods may not be limited to a single mosquito-borne virus, but have the potential to be applied in the detection of different arboviruses. This is particularly important in areas where dengue fever, chikungunya fever, Zika virus disease and other mosquito-borne viral diseases may be epidemic during the same period, because infections caused by mosquito-borne viruses often share similar clinical manifestations, such as fever, rash, headache, myalgia and arthralgia, making clinical diagnosis difficult in the early stage (Paixão et al., 2018; Silva MMO. et al., 2019). Actually, dengue remains one of the most important mosquito-borne viral diseases worldwide, which has been recognized as one of the top ten threats to global health by the WHO in 2019, and its large disease burden has promoted continuous development of rapid molecular diagnostic methods (Bhatt et al., 2013; Scheres and Kuszewski, 2019; Zhang et al., 2025). Meanwhile, the re-emergence and epidemic of non-DENV arboviruses, such as ZIKV, CHIKV and WNV, also require rapid and reliable laboratory evidence for differential diagnosis and public health surveillance (Adam and Jassoy, 2021; Madere et al., 2025). However, the non-DENV subgroup in our analysis included different viruses and a relatively limited number of data sets, and several data sets reported zero false positives, reminding us that the high specificity in this subgroup should be interpreted cautiously. In this case, our results suggest that RPA-based assays have broad application potential for mosquito-borne virus detection, but more prospective studies with larger sample sizes and different viral targets are still needed to further confirm their diagnostic performance in real-world settings.

Although RPA-based assays showed high diagnostic accuracy in our study, we have to admit that their practical application in real-life clinical or public health settings is still limited. For mosquito-borne virus diagnosis, RT-qPCR or real-time RT-PCR remains the most commonly used molecular method in laboratories. For example, the CDC DENV-1–4 real-time RT-PCR assay has been developed and evaluated as an *in-vitro* diagnostic platform for dengue virus detection and serotyping, and several commercial RT-PCR-based assays for arboviruses have also been reported in recent studies (Santiago et al., 2013; Waggoner et al., 2013; Johnson et al., 2016; Kingwara et al., 2025). In contrast, most RPA-based methods for detecting mosquito-borne viruses remain at an early stage, such as methodological development and validation using limited clinical samples, and they lack large-scale clinical validation (Patel et al., 2016; Vasileva Wand et al., 2018; Varghese et al., 2023; Hueso et al., 2025). This may be the main reason why RPA has not yet been widely used in medical institutions or public health laboratories, despite its rapid and simple amplification characteristics. Therefore, similar to other newly developed molecular diagnostic methods, several practical problems need to be solved before RPA-based assays can be widely used in real-world settings.

Firstly, the processing of clinical samples still needs to be developed and improved. Especially for RNA viruses, nucleic acid extraction is one of the most important steps, and it may affect the total testing time and the stability and reliability of the final results. Secondly, the reaction conditions, primer design and result interpretation of RPA-based assays still need to be further unified, because non-specific amplification or contamination may lead to false-positive results. Thirdly, more clinical samples should be used to test such method in different sample types, different virus types and in different epidemic conditions. What’s more, cost control, personnel training and integration with portable devices should also be considered before practical application. Therefore, future studies should not only focus on analytical performance, but also test their actual performance in real clinical and public health institutions. For example, more prospective studies are needed to evaluate whether these methods can still work well in hospitals, public health laboratories and outbreak investigation during the epidemic. These studies will help us evaluate whether RPA-based assays can maintain good performance outside the laboratory and can be truly implemented in practice for rapid diagnosis and public health response of mosquito-borne viral diseases.

Finally, several limitations should also be seriously considered when interpreting the results of the present study. First, although Deeks’ funnel plot asymmetry test did not show significant publication bias in our study, the number of included studies was still limited. Therefore, the absence of significant publication bias does not mean that publication bias completely did not exist (Bürkner and Doebler, 2014). What’s more, our QUADAS-2 assessment showed that several studies had high or unclear risk of bias in aspect of patient selection and index test. For example, some studies did not clearly report whether the samples were collected consecutively or randomly. Meanwhile, most studies did not clearly state how the operators interpreted the results produced by the RPA-based assay. These biases may make the diagnostic performance of RPA-based methods for detecting mosquito-borne viruses look better than that in actual clinical or public health practice. Similar to other diagnostic meta-analyses, studies with limited included researches, selected samples, or unclear interpretation of results may lead to an overestimated evaluation of diagnostic accuracy, which should be prudent when summarizing the conclusions of the meta-analyses (Sterne et al., 2011; Whiting et al., 2011).

Although RPA-based assays showed high pooled sensitivity and specificity in our study, most included studies were still at their laboratory research phase or clinical validation studies with limited sample sizes. For example, some data sets contained few positive samples, while several data sets reported zero false-positive results, which may influence the stability of the pooled estimates, especially the specificity and PLR. Therefore, the excellent diagnostic performance observed in our study should not be simply explained as the actual performance of RPA-based assays in all real-world settings. In addition, significant heterogeneity was observed in sensitivity in our analysis. Although the DOR-based heterogeneity analysis did not show significant heterogeneity, DOR combines sensitivity and specificity into a single indicator, and may mask the variability of sensitivity or specificity across data sets. Therefore, the variation in individual sensitivity and specificity estimates should also be noticed when interpreting the pooled results. These results also reminded us that the diagnostic performance of RPA-based assays may be influenced by some factors, such as virus type, sample size, sample source, assay design and study quality. Hence, more large-scale prospective studies are still needed to confirm whether these RPA-based methods can exhibit similar performance in clinical diagnosis. Therefore, in order to explain and evaluate our research results more rigorously, we further performed a leave-one-out sensitivity analysis to evaluate the robustness of our findings. Our results showed that after excluding each individual data set one at a time, the pooled sensitivity ranged from 0.946 to 0.962, while the pooled specificity ranged from 0.989 to 0.993. The PLR ranged from 86.000 to 137.286, NLR from 0.038 to 0.055, and DOR from 1575.074 to 3495.505. The changes were relatively small, indicating that the overall diagnostic performance was relatively stable and was not influenced by any single data set. Taken together, this result suggested that our findings were robust despite the presence of some methodological limitations in our included studies.

Last but not least, it should be noted that the present study mainly focused on the diagnostic accuracy of RPA-based assays, including sensitivity, specificity, likelihood ratios and AUC. Some practical indicators, such as detection time, limit of detection (LOD), cost and operational complexity, were not summarized in our study. This is because these indicators were not reported in a consistent way among the included studies. For example, some studies reported the amplification time only, while some studies may also include the time for sample processing or nucleic acid extraction. Similarly, the limit of detection was reported in different forms, such as copy number, RNA concentration or virus concentration, and some studies did not report these indicators clearly. Therefore, it was difficult to perform a reliable quantitative analysis on these practical indicators. However, this does not weaken the main value of our study. Previous studies have suggested that RPA has the advantages of rapid amplification, simple operation and low equipment requirement, and has potential value for point-of-care testing and emergency diagnosis (Daher et al., 2016; Hueso et al., 2025). Our results showed that RPA-based assays had high pooled sensitivity and specificity for mosquito-borne virus detection, suggesting that this method has good diagnostic accuracy. This finding can provide useful evidence for clinicians, laboratory workers and public health institutions when they consider whether RPA-based assays are worthy of further local validation or practical application. In other words, the present study answers the basic question of whether RPA-based assays are accurate enough for mosquito-borne virus detection. Based on this evidence, future studies should further evaluate whether these methods are also fast enough, simple enough and stable enough in real clinical and public health work. Such studies will be important for promoting the development of RPA-based assays from research methods to practical tools for rapid diagnosis and public health response.

Overall, although our sensitivity analysis suggested the robustness of the results, the small number of included studies, methodological limitations, and limited real-world practice remind us that the conclusions of the present study should still be interpreted with caution. Nevertheless, the high pooled sensitivity, specificity, PLR, NLR and AUC indicated that RPA-based assays had good diagnostic accuracy for mosquito-borne virus detection. When there is an outbreak of mosquito-borne viral diseases, early and accurate diagnosis remains important for case identification, epidemiological investigation and vector control. Therefore, RPA-based methods may provide a powerful and reliable technical option for rapid diagnosis and public health response. However, before such methods are widely implemented in practice, more high-quality studies with larger sample sizes, more rigorous study design and real clinical or public health settings are still required to confirm their real-world performance and practical value.

## Conclusion

In summary, our study showed that RPA-based methods exhibited good diagnostic accuracy for mosquito-borne virus detection, with a pooled sensitivity of 0.96 and specificity of 0.99. The high AUC, PLR and low NLR further suggested that this method has potential value for rapid case identification and public health response. Nevertheless, the small number of included studies, methodological limitations and limited real-world evidence warrant cautious interpretation of the results. Future studies with larger sample sizes, more rigorous study design and real clinical or public health settings are still necessary to validate the practical feasibility and application value of RPA-based methods for mosquito-borne virus detection.

## Data Availability

The original contributions presented in the study are included in the article/**Supplementary Material**, further inquiries can be directed to the corresponding author/s.

## Glossary

AUC: Area under the curve
CDC: Centers for Disease Control and Prevention
CHIKV: Chikungunya virus
CI: Confidence interval
CrI: Credible interval
CRISPR: Clustered regularly interspaced short palindromic repeats
DENV: Dengue virus
DNA: Deoxyribonucleic acid
DOR: Diagnostic odds ratio
ELISA: Enzyme-linked immunosorbent assay
FN: False negative
FP: False positive
IgG: Immunoglobulin G
IgM: Immunoglobulin M
LAMP: Loop-mediated isothermal amplification
LOD: Limit of detection
MEDLINE: Medical Literature Analysis and Retrieval System Online
NLR: Negative likelihood ratio
NS1: Non-structural protein 1
PCR: Polymerase chain reaction
PICOS: Population, Intervention, Comparator, Outcomes and Study design
PLR: Positive likelihood ratio
POCT: Point-of-care testing
PROSPERO: International Prospective Register of Systematic Reviews
QUADAS-2: Quality Assessment of Diagnostic Accuracy Studies-2
RNA: Ribonucleic acid
RPA: Recombinase polymerase amplification
RT-LAMP: Reverse transcription loop-mediated isothermal amplification
RT-PCR: Reverse transcription polymerase chain reaction
RT-qPCR: Reverse transcription quantitative polymerase chain reaction
RT-RPA: Reverse transcription recombinase polymerase amplification
SROC: Summary receiver operating characteristic
TN: True negative
TP: True positive
WHO: World Health Organization
WNV: West Nile virus
ZIKV: Zika virus
