# Supplementary material for: Diagnostic accuracy of recombinase polymerase amplification-based assays for mosquito-borne virus detection in clinical samples: a systematic review and meta-analysis
Source: Front Cell Infect Microbiol. 2026 Sep 3;16:1911702. doi: 10.3389/fcimb.2026.1911702 (PMC13581721; doi:10.3389/fcimb.2026.1911702)
Supplement: Supplementary file 1 [file Table1.docx]

**Supplementary Table S1. Additional conservative sensitivity analysis retaining only one Zhong_2025 serotype-specific data set at a time**

| Retained Zhong_2025 data set | No. of data sets included | Pooled sensitivity | 95% CrI | Pooled specificity | 95% CrI |
| --- | --- | --- | --- | --- | --- |
| Zhong_2025-1 | 10 | 0.957 | 0.913–0.987 | 0.996 | 0.989–1.000 |
| Zhong_2025-2 | 10 | 0.951 | 0.904–0.984 | 0.996 | 0.987–1.000 |
| Zhong_2025-3 | 10 | 0.948 | 0.899–0.983 | 0.997 | 0.988–1.000 |
| Zhong_2025-4 | 10 | 0.957 | 0.913–0.987 | 0.996 | 0.989–1.000 |
